# IDH2/R140Q mutation confers cytokine-independent proliferation of TF-1 cells by activating constitutive STAT3/5 phosphorylation

**DOI:** 10.1186/s12964-023-01367-y

**Published:** 2024-02-12

**Authors:** Jie Yang, Jiao Chen, Jingjie Chang, Xiaoyan Sun, Qingyun Wei, Xueting Cai, Peng Cao

**Affiliations:** 1 Jiangsu Provincial Medical Innovation Center, Affiliated Hospital of Integrated Traditional Chinese and Western Medicine, Nanjing University of Chinese Medicine, Nanjing, 210028 China; 2 The Quzhou Affiliated Hospital of Wenzhou Medical University, Quzhou People’s Hospital, Quzhou, 324000 China; 3 Zhenjiang Hospital of Chinese Traditional and Western Medicine, Zhenjiang, 212002 China

**Keywords:** Acute myeloid leukemia, Isocitrate dehydrogenase 2, R140Q mutation, Cytokine-independent proliferation, Signal transducer and activator of transcription 3/5

## Abstract

**Background:**

R140Q mutation in isocitrate dehydrogenase 2 (IDH2) promotes leukemogenesis. Targeting IDH2/R140Q yields encouraging therapeutic effects in the clinical setting. However, therapeutic resistance occurs in 12% of IDH2/R140Q inhibitor treated patients. The IDH2/R140Q mutant converted TF-1 cells to proliferate in a cytokine-independent manner. This study investigated the signaling pathways involved in TF-1(R140Q) cell proliferation conversion as alternative therapeutic strategies to improve outcomes in patients with acute myeloid leukemia (AML) harboring IDH2/R140Q.

**Methods:**

The effects of IDH2/R140Q mutation on TF-1 cell survival induced by GM-CSF withdrawal were evaluated using flow cytometry assay. The expression levels of apoptosis-related proteins, total or phosphorylated STAT3/5, ERK, and AKT in wild-type TF-1(WT) or TF-1(R140Q) cells under different conditions were evaluated using western blot analysis. Cell viability was tested using MTT assay. The mRNA expression levels of GM-CSF, IL-3, IL-6, G-CSF, leukemia inhibitory factor (LIF), oncostatin M (OSM), and IL-11 in TF-1(WT) and TF-1(R140Q) cells were quantified via RT-PCR. The secretion levels of GM-CSF, OSM, and LIF were determined using ELISA.

**Results:**

Our results showed that STAT3 and STAT5 exhibited aberrant constitutive phosphorylation in TF-1(R140Q) cells compared with TF-1(WT) cells. Inhibition of STAT3/5 phosphorylation suppressed the cytokine-independent proliferation of TF-1(R140Q) cells. Moreover, the autocrine GM-CSF, LIF and OSM levels increased, which is consistent with constitutive STAT5/3 activation in TF-1(R140Q) cells, as compared with TF-1(WT) cells.

**Conclusions:**

The autocrine cytokines, including GM-CSF, LIF, and OSM, contribute to constitutive STAT3/5 activation in TF-1(R140Q) cells, thereby modulating IDH2/R140Q-mediated malignant proliferation in TF-1 cells. Targeting STAT3/5 phosphorylation may be a novel strategy for the treatment of AML in patients harboring the IDH2/R140Q mutation.

Video Abstract

**Supplementary Information:**

The online version contains supplementary material available at 10.1186/s12964-023-01367-y.

## Background

Acute myeloid leukemia (AML) is a malignant cancer that poses a major threat to human health. Mutation of arginine to glutamine at position 140(R140Q) of isocitrate dehydrogenase 2 (IDH2) is closely related to the tumorigenesis of AML, and approximately 10% of patients with AML carry the IDH2/R140Q mutation [1, 2]. Impaired differentiation and malignant proliferation are two hallmarks of IDH mutated AML [3].

IDH mutation results in the production of (R) enantiomer of 2-hydroxyglutarate [(R)-2-HG], which competes with α-ketoglutarate (α-KG) to bind to the α-KG-dependent epigenetic regulatory enzymes. This leads to abnormal histone and DNA methylation, which in turn impairs stem cell differentiation [4, 5]. Targeting IDH2/R140Q is the main strategy for the treatment of mutated AML and works by restoring leukemia cell differentiation. Our previous studies identified several selective inhibitors of IDH2/R140Q that induce cellular differentiation in leukemia cells [6, 7]. Although the approved IDH2/R140Q inhibitor enasidenib exhibits encouraging therapeutic effects in patients with IDH2/R140Q mutated AML [8], acquired resistance to enasidenib has been reported [9], and approximately 12% of enasidenib-treated patients developed IDH differentiation syndrome, a potentially lethal clinical entity [10]. Therefore, it is necessary to identify IDH2/R140Q inhibition-independent strategies for the treatment of IDH2/R140Q-mutated AML.

Malignant proliferation is an important hallmark of IDH-mutated AML. IDH2/R140Q or mutation of arginine to histidine at position 132 (R132H) of IDH1 promotes cytokine independence and blocks differentiation in human TF-1 erythroleukemia cells [3, 11]. The IDH mutant product (R)-2-HG is sufficient for promoting leukemogenesis in TF-1 cells, and tet methylcytosine dioxygenase 2 (TET2) is an important target of (R)-2-HG that mediates the transformation of IDH1/R132H-mutated TF-1 cells. Although (R)-2-HG is a competitive inhibitor of the TET2 enzyme, TET2 gene knockdown did not suffice to transform TF-1 cells to the same extent as IDH mutation [3]. Accordingly, the identification of other mechanisms for IDH mutant TF-1 transformation is necessary to establish new strategies for the treatment of IDH-mutated AML.

This study investigated the mechanisms underlying the transformation of an IDH2/R140Q mutant TF-1 cell line from the perspective of malignant proliferation to identify new potential targets for the treatment of IDH2-mutated AML.

## Methods

### Cell lines and cultures

The human erythroleukemia cell line TF-1 (cat. no. CRL-2003) and IDH2-mutated TF-1 isogenic cell line TF-1(R140Q) (cat. no. CRL2003IG) were purchased from American Type Culture Collection (Manassas, VA, USA) in 2018 and cultured in RPMI-1640 medium supplemented with 10% fetal bovine serum and 5 ng/mL recombinant human granulocyte-macrophage colony stimulating factor (GM-CSF). All cell lines were cultured in humidified atmosphere in a 5% CO_2_ incubator at 37 °C.

### Reagent and antibodies

AGI-6780 was purchased from the TargetMol company. C188-9, NSC74859, and STAT5-IN-1 were purchased from the MCE company. Antibodies targeting signal transducer and activator of transcription 3 (STAT3), phospho-STAT3(Tyr^705^), STAT5, phospho-STAT5(Tyr^694^), extracellular signal-regulated kinase ERK1/2, phospho-ERK1/2(Thr^202^/Tyr^204^), protein kinase B AKT, phospho-AKT(Ser^473^), B-cell lymphoma-extra large (Bcl-xL), poly (ADP-ribose) polymerase (PARP), β-actin and glyceraldehyde-3-phosphate dehydrogenase (GAPDH) were purchased from the Cell Signaling Technology (Danvers, MA, USA). Methyl thiazolyl tetrazolium (MTT) was purchased from Sigma-Aldrich (St.Louis, MO, USA). Human recombinant GM-CSF and erythropoietin (EPO) were purchased from Tocris Bioscience (Bristol, UK).

### Western blotting

The wild type TF-1 [TF-1/(WT)] and/or TF-1(R140Q) cells were treated with the indicated concentrations of GM-CSF or compounds for indicated time points, then the cells were collected and the total protein was prepared. Western blotting was performed as previously reported [12], with indicated primary antibodies and secondary antibodies. Membranes were then washed and scanned with an Odyssey infrared fluorescent scanner (LI-COR).

### Cytotoxicity assays

TF-1(WT) and TF-1(R140Q) cells were plated in triplicate in a 96-well plate at a density of 1 × 10^4^ cells with 100 µL culture medium per well in the absence or presence of indicated concentrations of the compounds for indicated time points. Cell viability was then determined using an MTT assay.

### Apoptosis assays

Apoptotic cells were identified using a fluorescein isothiocyanate (FITC) Annexin V Apoptosis Detection Kit (BD Biosciences, USA) following the manufacturer’s protocol. The cells were washed and incubated with binding buffer containing Annexin V-FITC and 7-aminoactinomycin D (7-ADD) in the dark at 25 ℃ for 15 min. Subsequently, the degree of apoptosis was analyzed using a FACScan laser flow cytometer (FACS Calibur: Becton Dickinson, USA). The data were analyzed using the CELL Quest software. 

### Differentiation assay

TF-1(WT) and TF-1(R140Q) cells were washed three times by PBS to remove GM-CSF. The cell lines were induced to differentiate with 50 ng/mL of EPO in the presence or absence of indicated concentrations of AGI-6780, C188-9 or NSC74859. After 7 days, cells were harvested and photographed.

### RNA extraction and real-time polymerase chain reaction (RT-PCR)

TF-1(WT) and TF-1(R140Q) cell lines were plated in triplicate in a 6-well plate at a density of 2 × 10^5^ cells with 2 mL culture medium per well in the absence or presence of indicated concentrations of compound for indicated time points; then the cells were collected, RNA was extracted using TRIzol reagent (Invitrogen, Carlsbad, CA, USA), then DNA was synthesized using reverse transcription reagent (Vazyme Biotechnology, Nanjing, China). RT-PCR was performed using ChamQ Universal SYBR qPCR Master Mix (Vazyme Biotechnology). The expression levels of GM-CSF, interleukin 3 (IL-3), IL-6, granulocyte colony-stimulating factor (G-CSF), oncostatin M (OSM), leukemia inhibitory factor (LIF), and IL-11 were determined using specific primers. GAPDH was used as an endogenous control. The data were analyzed for fold differences using the 2^−ΔΔCT^ method. The following primers were used to amplify the human gene sequences:

GM-CSF: 5’-TCCTGAACCTGAGTAGAGACAC-3’ (sense),

                5*’*- TGCTGCTTGTAGTGGCTGG-3’ (antisense);

IL-3:        5’-CAGACAACGCCCTTGAAGACA-3*’* (sense),

                5*’*-GCCCTGTTGAATGCCTCCA-3*’* (antisense);

G-CSF:    5*’*-GCTGCTTGAGCCAACTCCATA-3*’* (sense), 

                5*’*-GAACGCGGTACGACACCTC-3*’* (antisense);

IL-6:        5*’*-ACTCACCTCTTCAGAACGAATTG-3*’* (sense),

                5*’*-CCATCTTTGGAAGGTTCAGGTTG-3*’* (antisense);

LIF:         5*’*- CCAACGTGACGGACTTCCC-3*’* (sense),

                5*’*- TACACGACTATGCGGTACAGC-3*’* (antisense);

OSM:      5*’*-CACAGACTGGCCGACTTAGAG-3*’* (sense),

                5*’*-AGTCCTCGATGTTCAGCCCA-3*’* (antisense);

IL-11:      5*’*-CGAGCGGACCTACTGTCCTA-3*’* (sense),

                5*’*- GCCCAGTCAAGTGTCAGGTG-3*’* (antisense);

GAPDH:  5*’*-GGAGCGAGATCCCTCCAAAAT-3*’* (sense),

                5*’*-GGCTGTTGTCATACTTCTCATGG-3*’* (antisense).

### Enzyme-linked immunosorbent assay (ELISA)

The amounts of GM-CSF, LIF and OSM in the culture supernatants were measured by sandwich ELISA kits purchased from Multi Sciences. Absorbance at 450 nm was measured using an ELISA microplate reader.

### Statistical analysis

The experimental results presented in the figures are representative of three or more independent experiments. The data are presented as the mean values ± SD. Statistical comparisons between the groups were performed using one-way ANOVA. Values of *P* < 0.05 were considered to be statistically significant.

## Results

### The IDH2/R140Q mutant converts TF-1 proliferation into a cytokine-independent manner

**Fig. 1:**
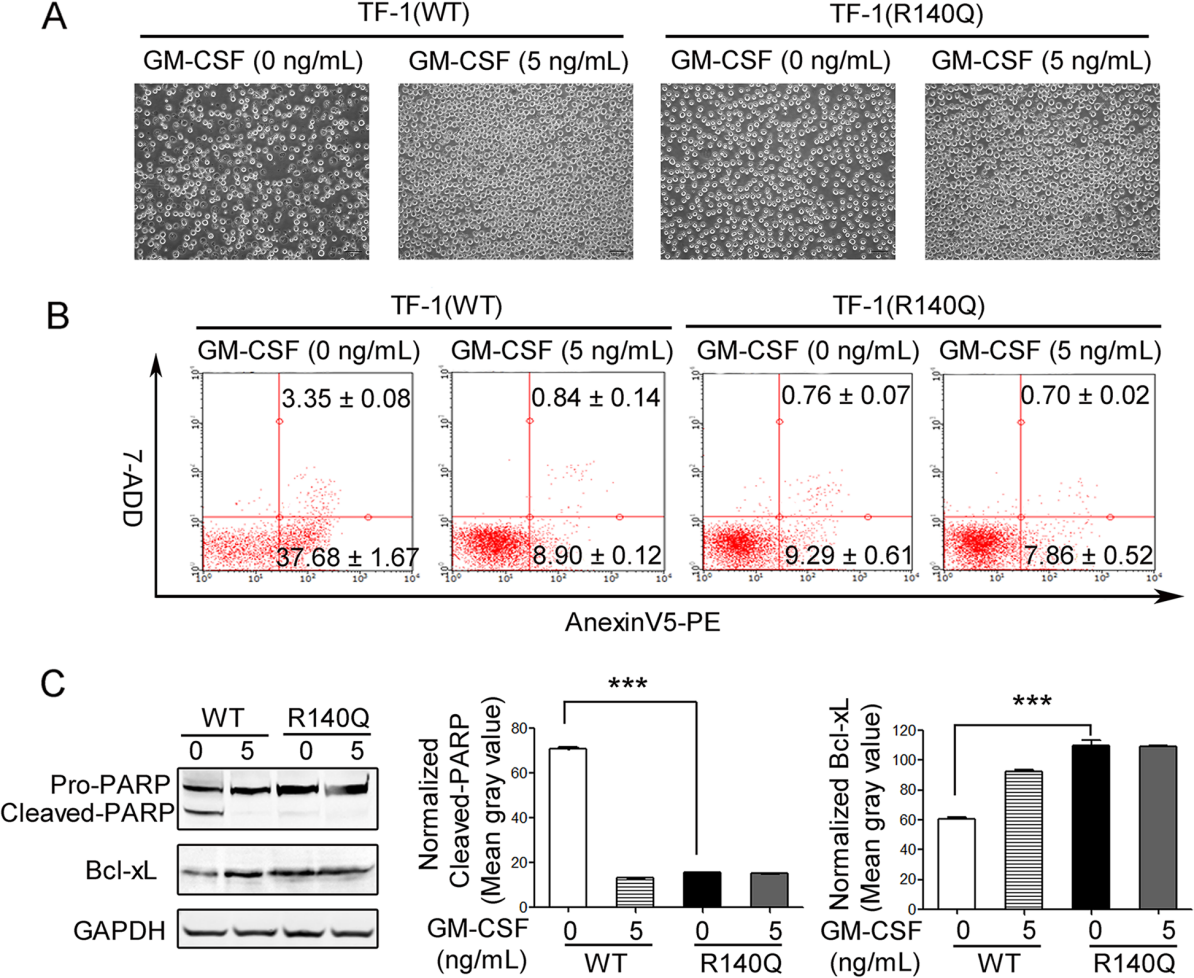
TF-1(R140Q) cells were resistant to apoptosis induced by GM-CSF withdrawal. After 24 h incubation in the absence or presence of 5 ng/mL GM-CSF, TF-1(WT) and TF-1(R140Q) cells reached different densities (**A**) and were subjected to flow cytometry assay for apoptosis (**B**). **C** TF-1(WT) and TF-1(R140Q) cells were cultured in the absence or presence of 5 ng/mL GM-CSF for 24 h, and PARP and Bcl-xL expression levels were analyzed. GAPDH was used as a loading control (left). Normalized mean gray values of cleaved-PARP (medium) and Bcl-xL (right) in TF-1(R140Q) cells were compared with TF-1(WT) cells in the absence of 5 ng/mL GM-CSF. ***P < 0.001 compared with TF-1(WT) cells. The dose of “0” group represents the vehicle control

The TF-1 cells are acute myeloid leukemia cells that depend on GM-CSF or IL-3 for cell survival and proliferation [13]. The IDH2/R140Q mutation confers TF-1 cells with cytokine-independent proliferation characteristics [11]. We further investigated the effects of IDH2/R140Q mutation on TF-1 cell survival upon GM-CSF withdrawal. TF-1(WT) and TF-1(R140Q) cells were cultured in the presence or absence of GM-CSF for 24 h, and apoptosis was analyzed. Cell proliferation was induced by GM-CSF in both TF-1(WT) and TF-1(R140Q) cells (Fig. 1A). Apoptosis was induced in TF-1(WT) cells, but not in TF-1(R140Q) cells, in the absence of GM-CSF (Fig. 1B). Correspondingly, cleavage of the apoptosis protein PARP and downregulation of the apoptosis inhibitor protein Bcl-xL only occurred in TF-1(WT) cells in the absence of GM-CSF (Fig. 1C). Collectively, these findings revealed that TF-1(R140Q) cells are resistant to apoptosis induced by GM-CSF withdrawal.

### The constitutive phosphorylation levels of STAT3(Tyr^705^) and STAT5(Tyr^694^) are increased significantly in TF-1(R140Q) cells

**Fig. 2:**
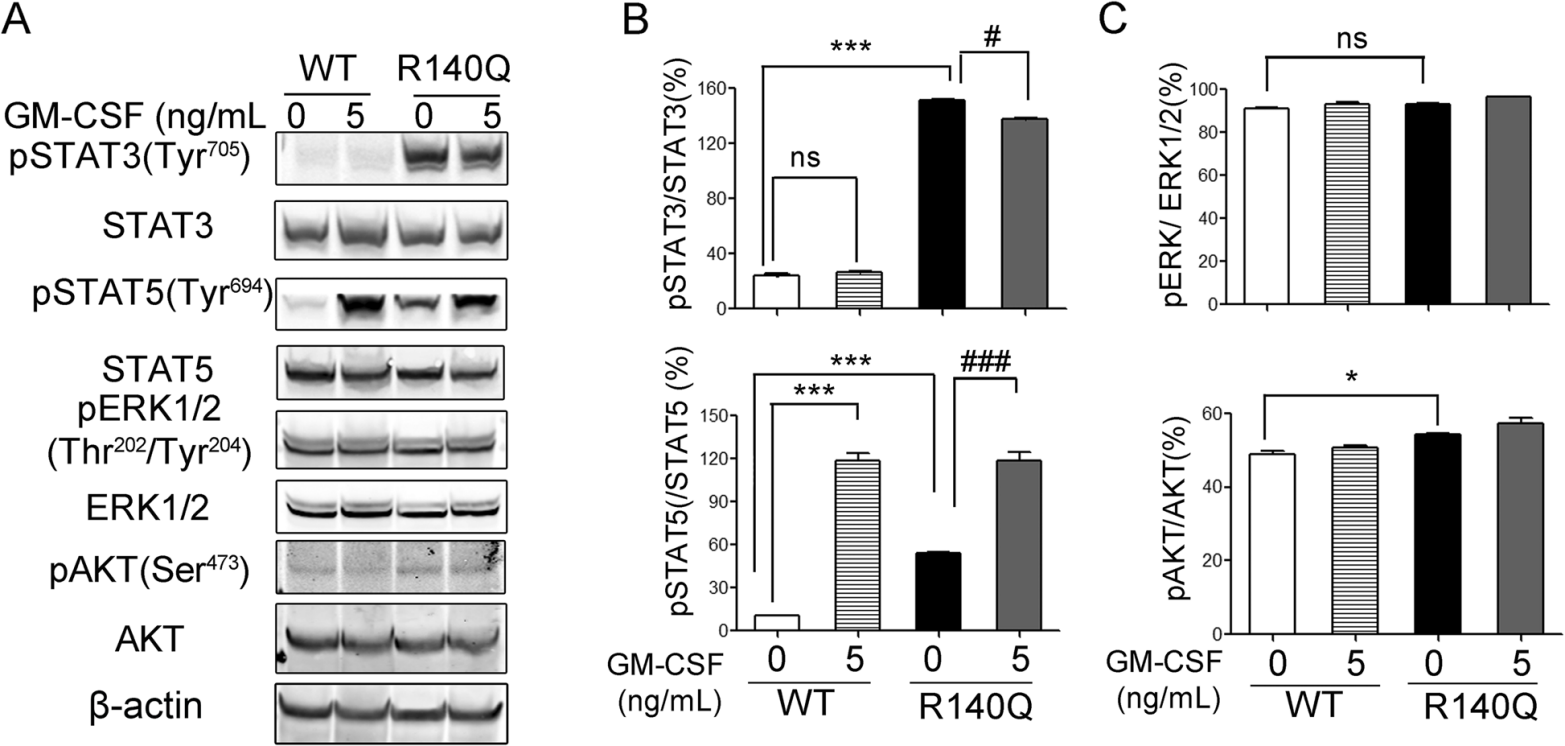
Aberrant constitutive activation of STAT3 and STAT5 with induced phospho-STAT3(Tyr^705^ ) and phospho-STAT5(Tyr^694^ ) levels in TF-1(R140Q) cells. **A** TF-1(WT) and TF-1(R140Q) cells were cultured in the absence or presence of 5 ng/mL GM-CSF for 24 h and subjected to western blotting for detection of the indicated proteins. **B**,** C** Ratios of the mean gray values of the indicated phosphorylated proteins to the mean gray values of the indicated total proteins and rations of values in TF-1(WT) and TF-1(R140Q) cells in the absence or presence of GM-CSF were analyzed. Representative results of three independent experiments are shown. Error bars are standard deviations. *P < 0.05, ***P < 0.001 compared with TF-1(WT) in the absence of GM-CSF; ^###^*P* < 0.001 compared with TF-1(WT) in the presence of GM-CSF; ns indicates no significance. The dose of “0” group represents the vehicle control

To identify the signaling pathways associated with the cytokine-independent proliferation of TF-1(R140Q) cells, we screened the activation state (phosphorylation) of signaling proteins critical for the survival and proliferation of TF-1 cells in the absence or presence of GM-CSF for 24 h (Fig. 2A). Our results demonstrated that constitutive STAT3 phosphorylation (Tyr^705^) is significantly increased in TF-1(R140Q) cells compared with TF-1(WT) cells, regardless of the presence or absence of GM-CSF (Fig. 2B). Moreover, the level of phospho-STAT5(Tyr^694^) is also significantly increased in TF-1(R140Q) cells compared with TF-1(WT) cells in the absence of GM-CSF (Fig. 2A and B). However, GM-CSF did not induce phosphorylation of STAT3(Tyr^705^) in TF-1(WT) cells, instead, it significantly induced phosphorylation of STAT5(Tyr^694^) (Fig. 2A and B). Additionally, in the absence of GM-CSF, the phospho-AKT (Ser^473^) was slightly increased in TF-1(R140Q) cells compared with TF-1(WT) cells, but the increased level is far less than phospho-STAT3 (Tyr^705^) and phospho-STAT5 (Tyr^694^). There was no significant change in phospho-ERK1/2(Thr^202^/Tyr^204^) between TF-1(WT) and TF-1(R140Q) cells, regardless of the presence or absence of GM-CSF (Fig. 2A and C).

### AGI-6780 inhibits phospho-STAT3(Tyr^705^) and phospho-STAT5(Tyr^694^) activation, and suppresses GM-CSF-independent TF-1(R140Q) cell proliferation

**Fig. 3:**
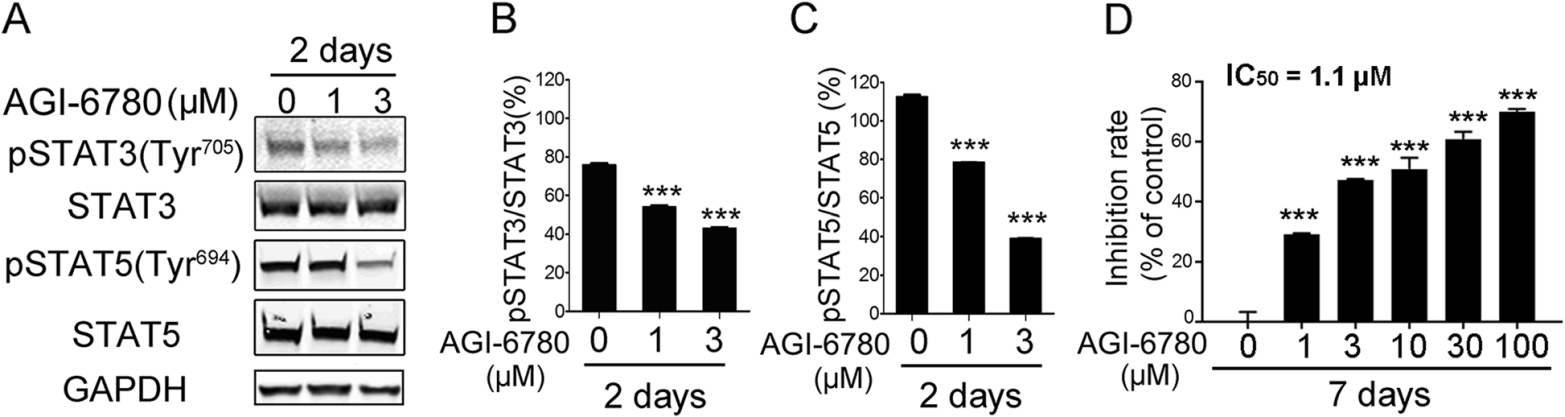
Effects of AGI-6780 on STAT3 and STAT5 activation in TF-1(R140Q) cells. **A** TF-1(R140Q) cells were treated with AGI-6780 for 2 days and subjected to western blotting for detection of the indicated proteins. GAPDH was used as a loading control. Ratios of the mean gray value of phospho-STAT3 (Tyr^705^) to the mean gray value of STAT3 (**B**), ratio of the mean gray value of pSTAT5 (Tyr^694^) to the mean gray value of STAT5 (**C**) and ratios in the treated group to the 0 µM of AGI-6780 group were analyzed. ***P < 0.001 compared with 0 µM of AGI-6780. **D** TF-1(R140Q) cells were treated with AGI-6780 for 7 days in the absence of GM-CSF, and cell viability was assayed by the MTT method. The inhibition rate was evaluated. The results are repetitive of three independent experiments with similar results, and error bars indicates standard deviations. ***P < 0.001 compared with 0 µM of AGI-6780 for 7 days. The dose of “0” group represents the vehicle control

To investigate the effect of IDH2/R140Q inhibition on phospho-STAT3(Tyr^705^) and phospho-STAT5(Tyr^694^) levels, TF-1(R140Q) cells were treated with the IDH2/R140Q inhibitor AGI-6780. The results showed that AGI-6780 treatment significantly decreased constitutive phospho-STAT3(Tyr^705^) (Fig. 3A and B) and phospho-STAT5(Tyr^694^) levels (Fig. 3A and C), and inhibited the cytokine-independent proliferation of TF-1(R140Q) cells with IC_50_ of 1.1 µM. (Fig. 3D).

### STAT3 and STAT5 inhibitors block cytokine-independent proliferation of TF-1(R140Q) cells

**Fig. 4:**
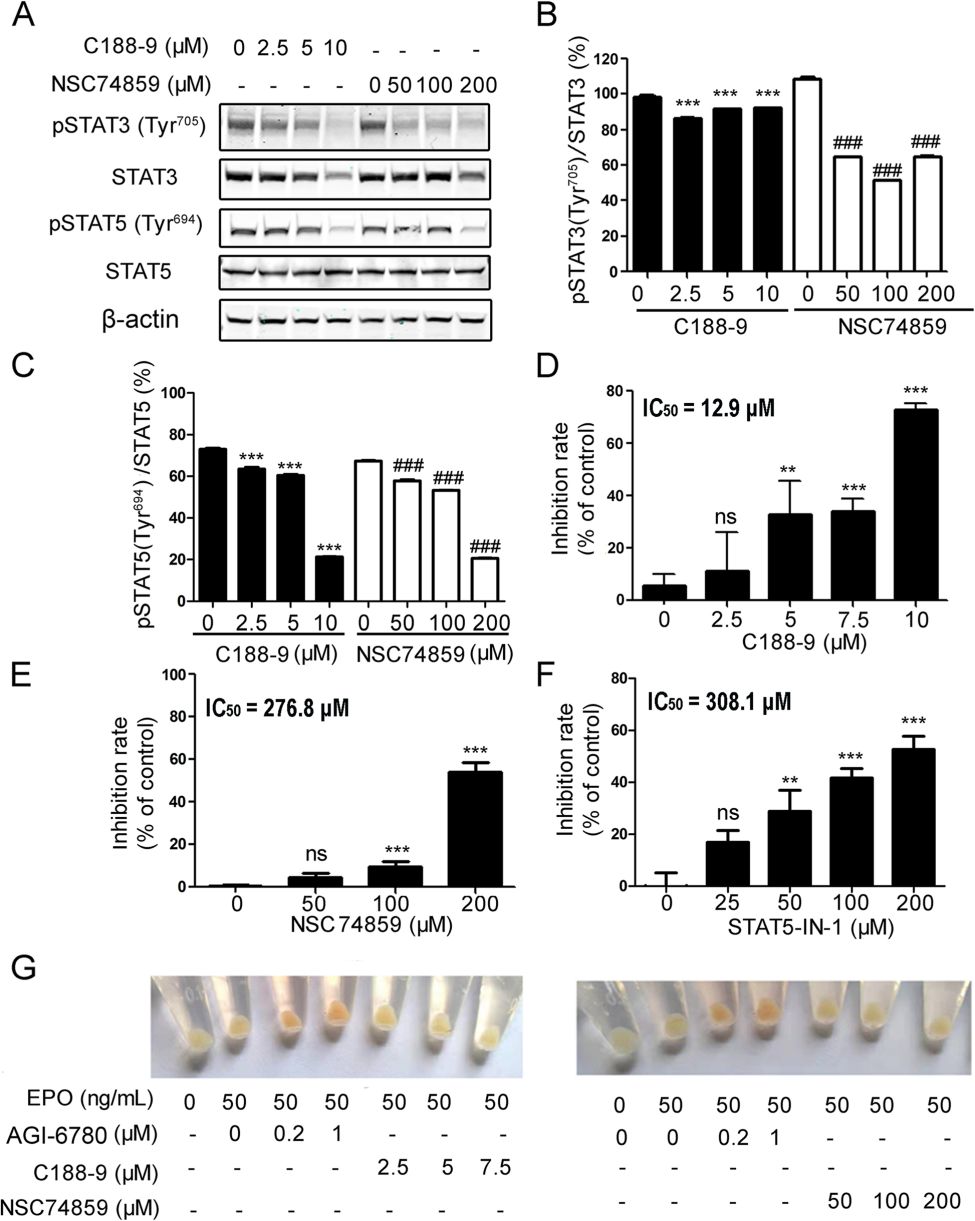
STAT3 and STAT5 inhibitors suppressed cytokine-independent TF-1(R140Q) cell proliferation. **A** TF-1(R140Q) cells were treated with C188-9 or NSC74859 for 24 h and subjected to western blotting for detection of the indicated proteins. β-Actin was used as a loading control. **B** The ratios of the mean gray value of phospho-STAT3(Tyr^705^) to the mean gray value of STAT3, (**C**) the ratios of the mean gray value of phospho-STAT5(Tyr^694^) to the mean gray value of STAT5 were calculated and the ratios of the treated groups to the control group were analyzed. The results represent three independent experiments with similar results, and the error bars indicate standard deviations. ***P < 0.001 compared with the control (0 µM C188-9). ^###^*P* < 0.001 compared with the control (0 µM NSC74859). TF-1(R140Q) cells were treated with C188-9 (**D**), NSC74859 (**E**) or STAT5-IN-1 (**F**) for 3 days in the absence of GM-CSF, and cell viability was evaluated using MTT assays. The inhibition rate of three independent experiments with similar results, and error bars mark indicate standard deviations. **P < 0.01, ***P < 0.001 compared with the vehicle control. **G** TF-1(R140Q) cells were induced with 50 ng/mL EPO to differentiate for 7 days in the presence of AGI-6780 (0.2, 1µM), C188-9 (2.5, 5, 7.5 µM) or NSC74859 (50, 100, 200 µM), respectively. Afterwards, the cells were collected, and color change was photographed. The dose of “0” and/or “-” group represents the vehicle control

To investigate the effects of aberrant phospho-STAT3(Tyr^705^) and phospho-STAT5(Tyr^694^) activation on cytokine-independent survival and proliferation of TF-1(R140Q) cells, the STAT3 inhibitors (C188-9, NSC74859) and STAT5 inhibitor STAT5-IN-1 were used to evaluate the cytokine-independent proliferation of TF-1 (R140Q) cells. The concentrations of the inhibitors we used were referred to the previous published papers [14–16]. We found that both C188-9 and NSC74859 decreased the levels of phospho-STAT3(Tyr^705^) (Fig. 4A and B) in TF-1(R140Q) cells. C188-9 and NSC74859 also decreased the level of phospho-STAT5 (Tyr^694^) to some degree (Fig. 4A and C). C188-9, NSC74859 and STAT5-IN-1 blocked the cytokine-independent proliferation of TF-1(R140Q) cells in a concentration-dependent manner with IC_50_ of 12.9 µM, 276.8 µM and 308.1 µM respectively (Fig. 4D, E and F). The two STAT3 inhibitors were used to investigate whether aberrant phospho-STAT3(Tyr^705^) could be involved in the differentiation block of TF-1(R140Q) cells. The results showed that C188-9 and NSC74859 did not restore the EPO-induced differentiation of TF-1(R140Q) cells at the used concentration (Fig. 4G). In contrast, AGI-6780 restored differentiation as demonstrated by the change in red color (Fig. 4G). These results indicated that the aberrant phosphorylation of STAT3(Tyr^705^) and STAT5 (Tyr^694^) in TF-1(R140Q) cells is involved in cytokine-independent survival and proliferation, but not differentiation.

### Autocrine GM-CSF and the IL-6 family members LIF and OSM facilitate the cytokine-independent proliferation of TF-1(R140Q) cells

**Fig. 5:**
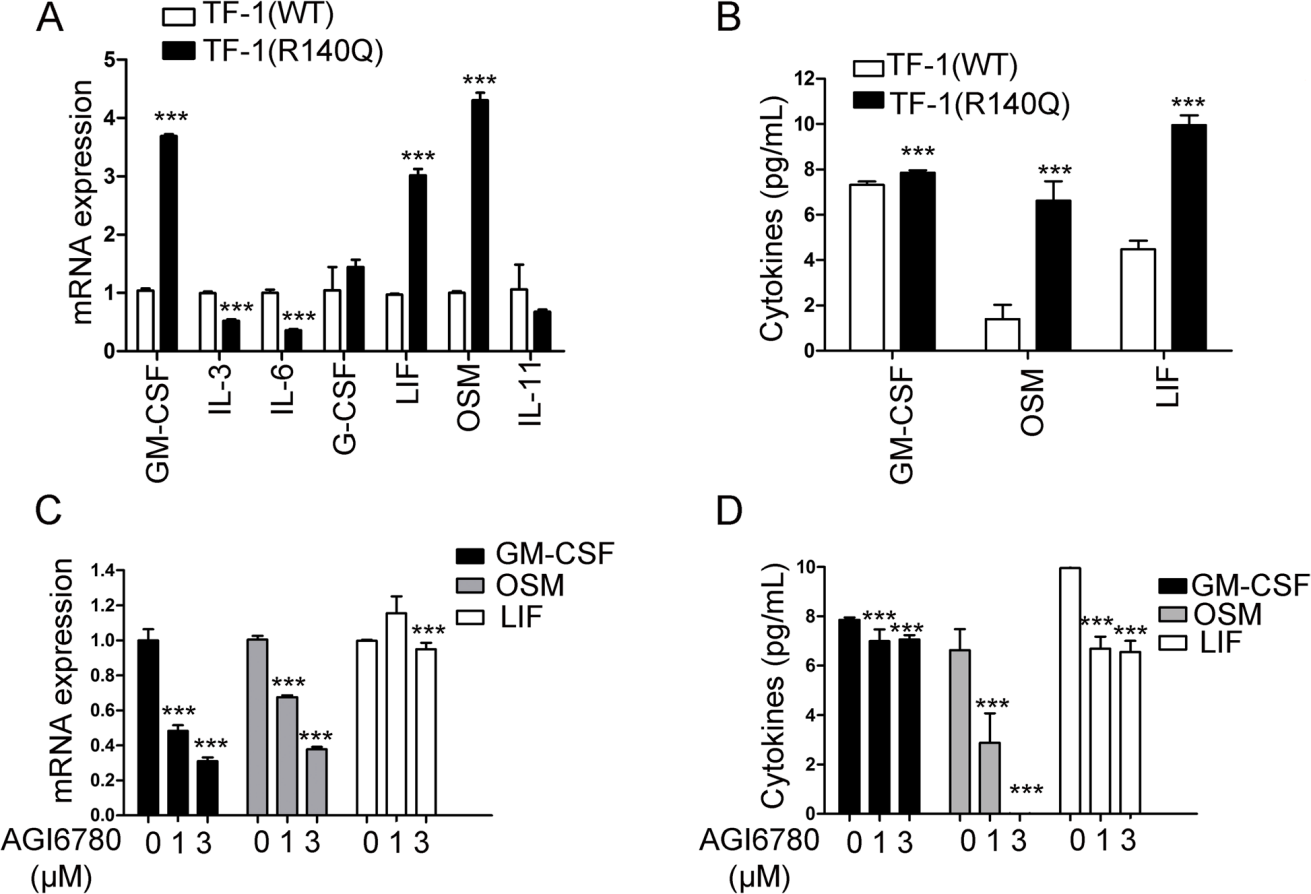
Autocrine GM-CSF, OSM, and LIF in TF-1(R140Q) cells determined the cytokine-independent transformation of TF-1(R140Q) cells. **A** mRNA expression levels of GM-CSF, IL-3, IL-6, G-CSF, LIF, OSM, and IL-11 in TF-1(WT) and TF-1(R140Q) cells in the absence of GM-CSF for 24 h. ***P < 0.001, compared with TF-1(WT) cells. **B** Secretion of GM-CSF, OSM, and LIF from TF-1(WT) and TF-1(R140Q) cells in the absence of GM-CSF for 24 h. ***P < 0.001, compared with TF-1(WT) cells. **C** Effects of AGI-6780 treatment on the mRNA expression of GM-CSF, OSM, and, LIF in TF-1(R140Q) cells in the absence of GM-CSF for 24 h. ***P < 0.001 compared with the control (0 µM AGI-6780). **D** Effects of AGI-6780 on GM-CSF, OSM, and LIF protein secretion in TF-1(R140Q) cells in the absence of GM-CSF for 24 h. ***P < 0.001 compared with the control (0 µM of AGI-6780). The dose of “0” group represents the vehicle control

Next, we evaluated the cause of aberrant activation of phospho-STAT3(Tyr^705^) and phospho-STAT5(Tyr^694^) in TF-1(R140Q) cells. Because phospho-STAT5(Tyr^694^) was induced by GM-CSF in both TF-1(WT) and TF-1(R140Q) cells, phospho-STAT5(Tyr^694^) may be the key signaling protein mediating GM-CSF-dependent proliferation, and phospho-STAT5(Tyr^694^) was constitutively present in TF-1(R140Q) cells in the absence of GM-CSF. Therefore, we investigated the mRNA levels and secretion of GM-CSF in TF-1(R140Q) cells. We found that TF-1(R140Q) cells expressed higher levels of GM-CSF mRNA and protein than TF-1(WT) cells (Fig. 5A and B). The STAT3 phosphorylation is mainly dependent on IL-6 family members. Our results showed that the mRNA and secreted proteins levels of LIF and OSM (which are two members of IL-6 family cytokines) are significantly increased in TF-1(R140Q) cells compared with TF-1(WT) cells (Fig. 5A and B).

To verify whether the changes in GM-CSF, LIF, and OSM expression were related to IDH2/R140Q mutation in TF-1(R140Q) cells, we investigated the effect of AGI-6780 on the mRNA expression and protein secretion of GM-CSF, LIF, and OSM. We found that AGI-6780 treatment inhibited the increase in GM-CSF, LIF and OSM levels in TF-1(R140Q) cells (Fig. 5C and D).

## Discussion

Targeting IDH2/R140Q yields encouraging therapeutic effects in the clinical setting. However, therapeutic resistance and isocitrate dehydrogenase differentiation syndrome occur in some IDH2/R140Q inhibitor-treated AML patients [8–10]. Accordingly, novel therapeutic strategies for IDH2-mutated AML are urgently needed.

This study demonstrated the activation of STAT3/5 in TF-1 cells with the IDH2/R140Q mutation. Our findings, together with those from a previous study [11], confirmed that IDH2/R140Q mutation transform TF-1 cells proliferation into a cytokine-independent manner. Mutations in various epigenetic modifiers and the Janus kinase/STAT pathway are underlying causes of many cancers, particularly acute leukemia and lymphomas [17]. Constitutive STAT3 and STAT5 activation induced by active leukemic fusion proteins is sufficient for the transformation of hematopoietic precursor cells [18]. We observed aberrant constitutive phosphorylation of STAT5(Tyr^694^) and STAT3(Tyr^705^) in TF-1(R140Q) cells. Both phospho-STAT3/phospho-STAT5 inhibitors and IDH2/R140Q mutant inhibitors abolished the cytokine independence of TF-1(R140Q) cells, demonstrating that constitutive phospho-STAT5(Tyr^694^) and phospho-STAT3(Tyr^705^) play critical roles in malignant transformation of TF-1(R140Q) cells. Some previous studies also support the critical role of phospho-STAT3(Tyr^705^) in the tumorigenic features of IDH mutated cancer. The insulin-like growth factor 1 receptor/AKT/STAT3 signaling pathway is reported to be involved in IDH1/R132H-induced malignant transformation of the benign prostatic epithelium [19]. Kotredes et al. reported that active STAT3 was elevated in both U87MG and U251 cells that carry either IDH2/R140Q or IDH2/R172M mutants [20]. Constitutive STAT3 and STAT5 activities were found in 28% and 22% of patient with AML [21], and phospho-STAT3 levels in AML blasts are an independent prognostic factor for overall survival [22]. STAT5 inhibitor has been proposed in the treatment of fms-like tyrosine kinase 3 (FLT3), TET2, and IDH-mutated AML [23, 24]. STAT5 inhibition enhances the differentiation response to IDH1 and IDH2 inhibitors in primary human IDH-mutated AML cells [25]. Accordingly, these findings suggested that targeting phospho-STAT3(Tyr^705^) and phospho-STAT5(Tyr^694^) may be a potential novel therapy strategy for IDH2/R140Q-mutated AML.

Next, we investigated the mechanisms of constitutive STAT3 and STAT5 phosphorylation in TF-1(R140Q) cells. We found that STAT5(Tyr^694^) activation play critical roles in GM-CSF-dependent proliferation of TF-1 cells because GM-CSF induced the activation of STAT5(Tyr^694^) in both TF-1(WT) and TF-1(R140Q) cells. Similarly, GM-CSF has been reported to preferentially activate STAT5A in human peripheral blood monocytes, and STAT5A-deficient mice demonstrate defects in GM-CSF induced proliferation and gene expression [26, 27]. In this study, the mRNA and protein secretion levels of GM-CSF were higher in TF-1(R140Q) cells than in TF-1(WT) cells, suggesting that constitutive phospho-STAT5 was mediated by autocrine GM-CSF. STAT3 is activated by several cytokines, particularly the IL-6 family. Higher secretion levels of LIF and OSM were observed in TF-1(R140Q) cells than TF-1(WT) cells, suggesting that constitutive phospho-STAT3(Tyr^705^) activation is mediated by autocrine LIF and OSM. TF-1 cells also proliferate in response to IL-3, LIF and OSM [28]. The mRNA expression of another TF-1-dependent cytokines, IL-3, and other IL-6 family members, including IL-6, G-CSF, and IL-11, did not differ between TF-1(WT) and TF-1(R140Q) cell lines. Furthermore, consistent with the inhibition of phospho-STAT3(Tyr^705^) and phospho-STAT5(Tyr^694^) by AGI-6780, AG-I6780 also inhibited the secretion of GM-CSF, LIF, and OSM, supporting that GM-CSF, LIF, and OSM are the downstream effectors of the IDH2/R140Q protein in TF-1(R140Q) cells. Moreover, (R)-2-HG can stimulate stromal cells to secrete IL-6, IL-8, and complement 5a for enhancing the proliferation of IDH mutant AML cells via paracrine signaling [29]. Our findings suggest that IDH mutated AML cells sustain self-proliferation via autocrine signaling. Moreover, as IL-6, OSM, and LIF utilize gp130 as a signaling component for high-affinity receptor complexes [28], targeting gp130 to inhibit autocrine LIF and OSM signaling could be another strategy for IDH-mutated AML therapy.

Although we hypothesized that constitutive STAT3/STAT5 activation induced by autocrine GM-CSF, LIF and OSM contribute to IDH/R140Q-mutated TF-1 cell transformation in this study, there are some limitations. First, the effects of the IDH mutant were mainly attributed to (R)-2-HG production, however, we did not evaluate how (R)-2-HG selectively upregulates GM-CSF, LIF, and OSM expression in TF-1(R140Q) cells. LIF expression is known to be upregulated via epigenetic changes and upregulation of LIF is essential for the development of breast cancer via autocrine mechanisms [30]. Accordingly, investigations to assess the epigenetic enzymes that regulate LIF expression in TF-1(R140Q) cells are warranted. Second, the levels of GM-CSF, LIF, OSM, phospho-STAT3(Tyr^705^), and phospho-STAT5(Tyr^694^) should be investigated in AML patients with IDH2/R140Q mutant and IDH2/WT to provide evidence for targeting phospho-STAT5(Tyr^694^), and phospho-STAT3(Tyr^705^) in the treatment of IDH2-mutated AML.

## Conclusion

In summary, we discovered that constitutive STAT3(Tyr^705^) and STAT5(Tyr^694^) activation induced by autocrine GM-CSF, LIF, and OSM contribute to the cytokine-independent survival and proliferation of IDH2/R140Q-mutated TF-1 cells. Our study suggests that targeting phospho-STAT3(Tyr^705^) and phospho-STAT5(Tyr^694^) could have applications in the treatment of IDH2/R140Q-mutated AML.

### Supplementary Information

**Additional file 1:** **Figure 1. **TF-1(WT) and TF-1(R140Q) cells were cultured in the absence or presence of 5 ng/mL GM-CSF for 24 h, and PARP and Bcl-xL expression levels were analyzed by western blotting. GAPDH was used as a loading control. **Figure 2.** TF-1(WT) and TF-1(R140Q) cells were cultured in the absence or presence of 5 ng/mL GM-CSF for 24h and subjected to western blotting for detection of the indicated proteins. β-Actin was used as a loading control. **Figure 3. **TF-1(R140Q) cells were treated with AGI-6780 for 2 days and subjected to western blotting for detection of the indicated proteins. GAPDH was used as a loading control. **Figure 4. **TF-1(R140Q) cells were treated with C188-9 or NSC74859 for 24 h and subjected to western blotting for detection of the indicated proteins. β-Actin was used as a loading control. 

## Data Availability

The data supporting the conclusions of this article are included within this article and the supplementary information file. Further information is available from the corresponding author on reasonable request.

## Abbreviations

7-ADD: 7-Aminoactinomycin D
α-KG: α-ketoglutarate
AML: Acute myeloid leukemia
ATCC: American type culture collection
Bcl-2: B-cell lymphoma-2
Bcl-xL: B-cell lymphoma-extra large
EPO: Erythropoietin
ERK: Extracellular signal-regulated kinases
FITC: Fluorescein isothiocyanate
FLT3: fms-like tyrosine kinase 3
GAPDH: Glyceraldehyde-3-phosphate dehydrogenase
GM-CSF: Granulocyte-macrophage colony stimulating factor
HBG: Hemoglobinγ
IDH: Isocitrate dehydrogenase
JAK: Janus kinase
LIF: Leukemia inhibitory factor
MAPK: Mitogen-activated protein kinase
MTT: Methyl thiazolyl tetrazolium
OSM: Oncostatin M
PARP: Poly ADP ribose polymerase
R140Q: Arginine to glutamine mutation at position 140
**(**R)-2-HG: (R)-2-hydroxyglutarate
Ser: Serine
STAT: Signal transducer and activator of transcription
TBST: Tris-buffered saline and tween 20
TET2: Tet methylcytosine dioxygenase 2
Thr: Threonine
TYK2: Tyrosine kinase 2
Tyr: Tyrosine
WT: Wild type
