# Supplementary material for: IDH2/R140Q mutation confers cytokine-independent proliferation of TF-1 cells by activating constitutive STAT3/5 phosphorylation
Source: Cell Commun Signal. 2024 Feb 12;22:116. doi: 10.1186/s12964-023-01367-y (PMC10863291; doi:10.1186/s12964-023-01367-y)
Supplement: Supplementary file 2 — Additional file 1: Figure 1. TF-1(WT) and TF-1(R140Q) cells were cultured in the absence or presence of 5 ng/mL GM-CSF for 24 h, and PARP and Bcl-xL expression levels were analyzed by western blotting. GAPDH was used as a loading control. Figure 2. TF-1(WT) and TF-1(R140Q) cells were cultured in the absence or presence of 5 ng/mL GM-CSF for 24h and subjected to western blotting for detection of the indicated proteins. β-Actin was used as a loading control. Figure 3. TF-1(R140Q) cells were treated with AGI-6780 for 2 days and subjected to western blotting for detection of the indicated proteins. GAPDH was used as a loading control. Figure 4. TF-1(R140Q) cells were treated with C188-9 or NSC74859 for 24 h and subjected to western blotting for detection of the indicated proteins. β-Actin was used as a loading control. [file 12964_2023_1367_MOESM1_ESM.docx]

The raw western blotting images:


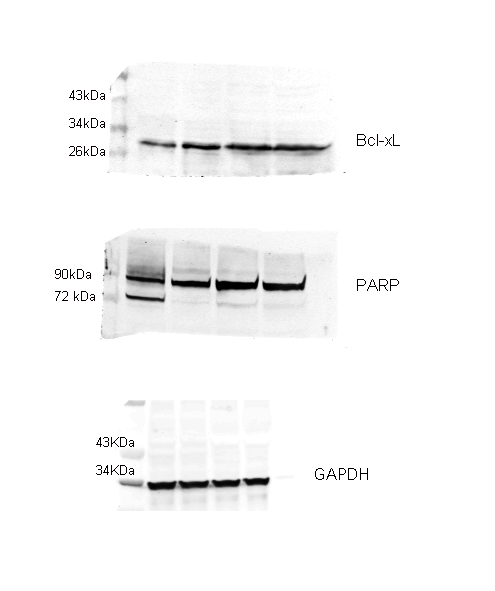


**Figure 1 (C)** TF-1(WT) and TF-1(R140Q) cells were cultured in the absence or presence of 5 ng/mL GM-CSF for 24h, and PARP and Bcl-xL expression levels were analyzed by western blotting. GAPDH was used as a loading control.


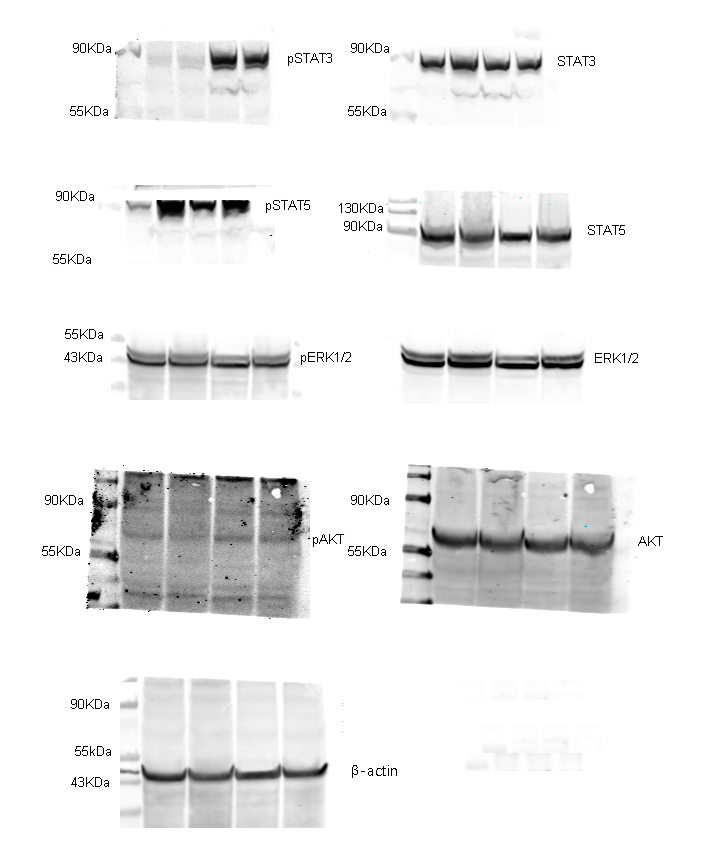


Figure 2 **(A)** TF-1(WT) and TF-1(R140Q) cells were cultured in the absence or presence of 5 ng/mL GM-CSF for 24h and subjected to western blotting for detection of the indicated proteins. β-Actin was used as a loading control.


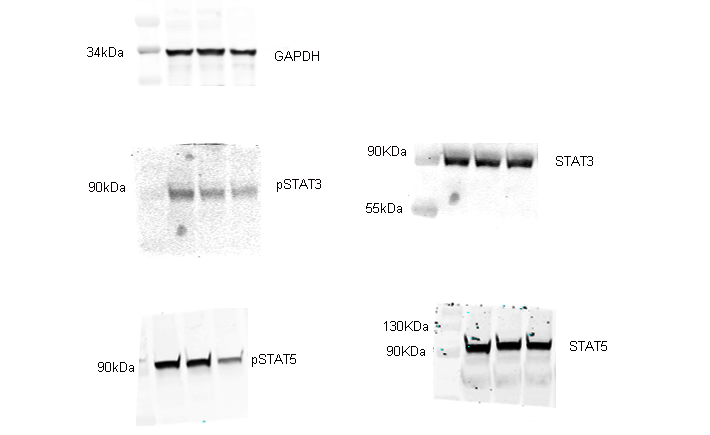


**Figure 3 (A)** TF-1(R140Q) cells were treated with AGI6780 for 2 days and subjected to western blotting for detection of the indicated proteins. GAPDH was used as a loading control.


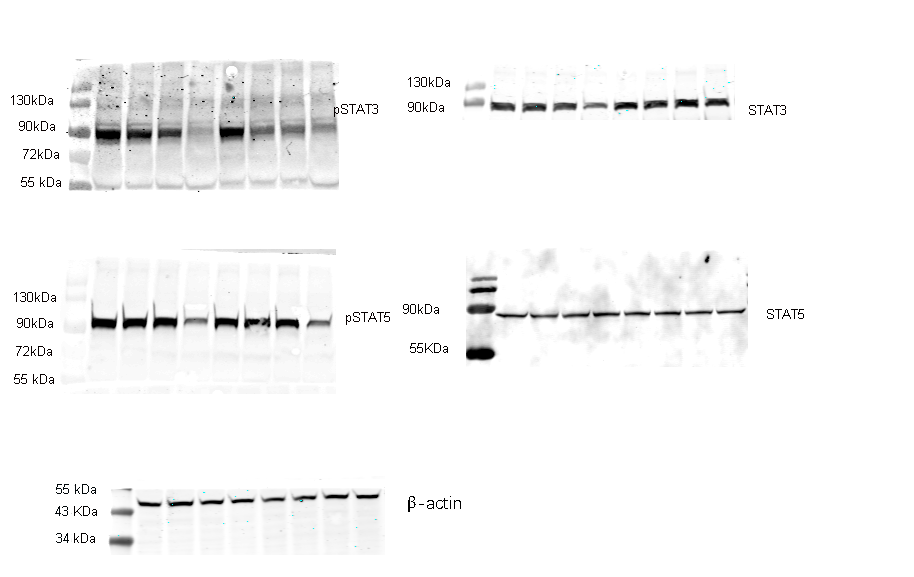


**Figure 4 (A)** TF-1(R140Q) cells were treated with C188-9 or NSC74859 for 24 h and subjected to western blotting for detection of the indicated proteins. β-Actin was used as a loading control.
